# The Greenhouse gas Observations of Biospheric and Local Emissions from the Upper sky (GOBLEU): a mission overview, instrument description, and results from the first flight

**DOI:** 10.1186/s13021-024-00273-1

**Published:** 2024-08-16

**Authors:** Hiroshi Suto, Akihiko Kuze, Ayako Matsumoto, Tomohiro Oda, Shigetaka Mori, Yohsuke Miyashita, Chiharu Hoshino, Mayumi Shigetoh, Fumie Kataoka, Yasuhiro Tsubakihara

**Affiliations:** 1 Japan Aerospace Exploration Agency (JAXA), Tsukuba, Japan; 2 ANA Holdings Inc., Tokyo, Japan; 3 Earth from Space Institute, Universities Space Research Association (USRA), Columbia, MD USA; 4 Department of Atmospheric and Oceanic Science, University of Maryland, College Park, MD USA; 5 Graduate School of Engineering, Osaka University, Suita, Osaka Japan; 6 JASTECS, Tokyo, Japan; 7 Remote Sensing Technology Center of Japan (RESTEC), Tokyo, Japan

**Keywords:** Net zero, Paris accord, 1.5degree, Greenhouse gas, Climate monitoring, Climate mitigation, Global stocktake

## Abstract

**Background:**

The Greenhouse gas Observations of Biospheric and Local Emissions from the Upper sky (GOBLEU) is a new joint project by Japan Aerospace Exploration Agency (JAXA) and ANA HOLDING INC. (ANAHD), which operates ANA flights. GOBLEU aims to visualizes our climate mitigation effort progress in support of subnational climate mitigation by collecting greenhouse gas (GHG) data as well as relevant data for emissions (nitrous dioxide, NO_2_) and removals (Solar-Induced Fluorescence, SIF) from regular passenger flights. We developed a luggage-sized instrument based on the space remote-sensing techniques that JAXA has developed for Japan’s Greenhouse gas Observing SATellite (GOSAT). The instrument can be conveniently installed on a coach-class passenger seat without modifying the seat or the aircraft.

**Results:**

The first GOBLEU observation was made on the flight from the Tokyo Haneda Airport to the Fukuoka Airport, with only the NO_2_ module activated. The collected high-spatial-resolution NO_2_ data were compared to that from the TROPOspheric Monitoring Instrument (TROPOMI) satellite and surface NO_2_ data from ground-based air quality monitoring stations. While GOBLEU and TROPOMI data shared the major concentration patterns largely driven by cities and large point sources, regardless of different observation times, we found fine-scale concentration pattern differences, which might be an indication of potential room for GOBLEU to bring in new emission information and thus is worth further examination. We also characterized the levels of NO_2_ spatial correlation that change over time. The quickly degrading correlation level of GOBLEU and TROPOMI suggests a potentially significant impact of the time difference between CO_2_ and NO_2_ as an emission marker and, thus, the significance of co-located observations planned by future space missions.

**Conclusions:**

GOBLEU proposes aircraft-based, cost-effective, frequent monitoring of greenhouse emissions by GOBLEU instruments carried on regular passenger aircraft. Theoretically, the GOBLEU instrument can be installed and operated in most commercially used passenger aircraft without modifications. JAXA and ANAHD wish to promote the observation technique by expanding the observation coverage and partnership to other countries by enhancing international cooperation under the Paris Agreement.

## Background

Under the Paris Agreement, countries submit their pledges toward the temperature goal in the form of Nationally Determined Contributions (NDCs), and their progress will be evaluated every five years from 2023 (Global Stocktake, GST). The first GST suggested that our climate mitigation effort is insufficient to achieve Paris agreement goal, and thus, we need further actions [1]. Science-based approaches are necessary to track and guide our actions to the second GST in 2028. The research community has explored the ways to utilize greenhouse gas (GHG) data collected from various observation platforms (e.g., ground, aircraft, and satellites) in order to support the evaluation at GST [2]. Over the past decade, GHG remote sensing has significantly advanced, matured, and started playing a key role in collecting GHG data for science [e.g., 3–12], and for climate mitigation monitoring applications [e.g., 13–15].

In 2020, the Japan Aerospace Exploration Agency (JAXA) and ANA HOLDINGS INC. (ANAHD) jointly launched a new project named Greenhouse gas Observations of Biospheric and Local Emissions from the Upper sky (GOBLEU) (https://www.eorc.jaxa.jp/GOSAT/ANAexp/index.html). In support of subnational climate mitigation efforts, GOBLEU collects GHG and other relevant data from regular passenger aircraft and visualizes our climate mitigation effort progress. GOBLEU aims to achieve the objective by combining JAXA’s GHG remote-sensing technique and experiences built on the GOSAT mission and ANA’s world-leading air passenger carrier capacity. Just a few years back, the COVID-19 pandemic hit the airline industry hard due to the travel restrictions implemented globally [16]. The number of passenger flights in 2020 dropped by 60% from 2019 [17]. Airlines have not carried passengers at full capacity for over two years, including the Olympic and Paralympic Games 2021 in Tokyo. However, GOBLEU turns " ghost " flight operations into action for climate monitoring and mitigation by carrying a GHG monitoring instrument on board passenger flights and contributing to sustainability.

JAXA and ANAHD have designed and developed an innovative carry-on size instrument suite that can be installed on two window seats and collects data through the cabin window, just like a passenger. The GOBLEU instruments are based on the same remote-sensing observation technique developed and used by the current state-of-the-art GHG observing GOSAT (2009-on, 3) and GOSAT-2 (2018-on, 6) satellites. The instrument suite is designed to collect carbon dioxide (CO_2_) data as well as additional variables, such as nitrogen dioxide (NO_2_) levels as a tracer of CO_2_ from fuel combustion and solar-induced fluorescence (SIF) as an indicator of the plant production (carbon removals) of the terrestrial biosphere (e.g., forests and other vegetated areas) [18, 19]. This is expected to enhance the ability to monitor carbon emissions and removals from major key emission sectors and provide GHG information relevant to climate mitigation tracking at various decision making and climate action levels. Data to be collected by GOBLEU and the derived GHG information are complementary to common GHG information, such as emission inventories. These are typically developed and updated on a relatively low frequency (often annually) with high latency (more than a year or two). GOBLEU expects to provide timely GHG information by promptly collecting high-resolution GHG data and emission and removal estimates with greater information granularity. The high-spatial-resolution data should provide GHG information to stakeholders at different subnational levels (e.g., states/prefectures, cities, the private sectors, and citizens).

GOBLEU should also provide direct technical and scientific implications to the synergic use of remotely sensed GHG and AQ data that is planned by future space GHG observing missions, such as Japan’s Global Observing SATellite for Greenhouse gases and Water cycle (GOSAT-GW; planned launch 2024) [20] and Europe’s Copernicus Carbon Dioxide Monitoring mission (CO2M; planned launch 2026) [21]. As recent studies [22–26] demonstrated, simultaneously collecting CO_2_ and NO_2_ data should enhance our ability to quantify anthropogenic GHG emissions. However, it is important to note that the previous studies have been based on data from different satellite platforms based on certain spatial and temporal colocation criteria or a campaign flight.

The first GOBLEU proof of concept flight occurred on the 26th of October 2020 during the global COVID pandemic. This manuscript describes the instrument, the first flight, and the results. We also discuss the current challenges and limitations as well as our plans.

## Methods

### GOBLEU monitoring instrument suite

**Fig. 1:**
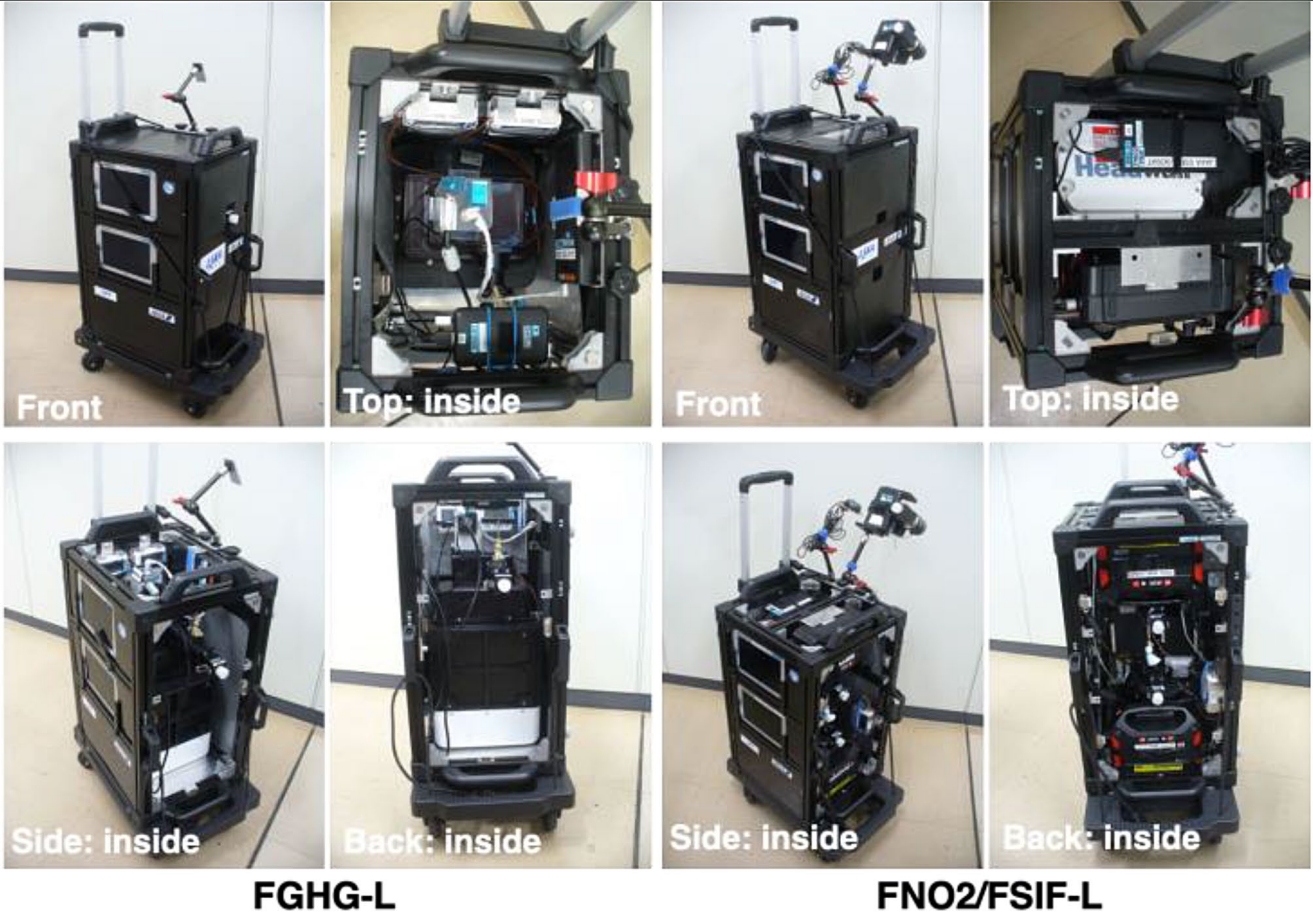
The GOBLEU monitoring instrument suite. The instrument suite consists of two modules: GHG observation (left) and NO_2_/SIF observation (right). These modules are connected by optical fiber from the window seat. The instrumental parameters, including the observation setting, differ between left-side and right-side seats. The instruments are labeled as “-L” or “-R” for their proper seat to avoid missetting

Figure 1 shows the GOBLEU monitoring instrument suite. Before the GOBLEU project launched, we evaluated the transmissivity of the cabin window using ASD FieldSpec4 (a wide-spectral range spectrometer). We confirmed that most cabin windows on ANA-operated aircraft transmit the reflected solar light spectra between 400 nm and 1650 nm sufficiently for the measurement, except for windows with an electronic shade. Given the spectral range, GOBLEU has chosen CO_2_ as the main target GHG. The instrument suite consists of three in-house customized imaging (grating) spectrometers coupled with 53 arrays of bundled optical fibers: Fiber-coupled NO_2_ imaging spectrometer (FNO2), Fiber-coupled SIF imaging spectrometer (FSIF), and Fiber-coupled Greenhouse gas imaging spectrometer (FGHG). Details of the spectrometers are summarized in Table 1. A common fore-optics relay is the solar light reflected on the Earth’s surface through the bundled optical fibers. Three instruments cover the spectral ranges in 420–490 nm for NO_2_, 670–780 nm for SIF, and 1560–1640 nm for CO_2_, with spectral sampling intervals of 0.03 nm, 0.05 nm, and 0.17 nm, respectively. These spectrometers are coupled with a 2-dimentional (2D)-CMOS camera (2048 × 2048) for NO_2_ and SIF and an 2D-InGaAs camera (640 × 512) for CO_2_ manufactured by Hamamatsu Photonics K.K. GOBLEU is a push broom hyperspectral imager, integrating spectroscopy and 2D-spatial mapping in one single system for each target species.

CO_2_, NO_2_, and SIF imaging spectrometers are packed in two carry-on luggage-sized boxes (one for NO_2_ and SIF observation and one for CO_2_ observation, 320 mm (W) x 380 mm (D) x 600 mm (H) per pack). They weigh less than 30 kg and collect high-spatial-resolution remote-sensing spectra of NO_2_, SIF, and CO_2_.

**Fig. 2:**
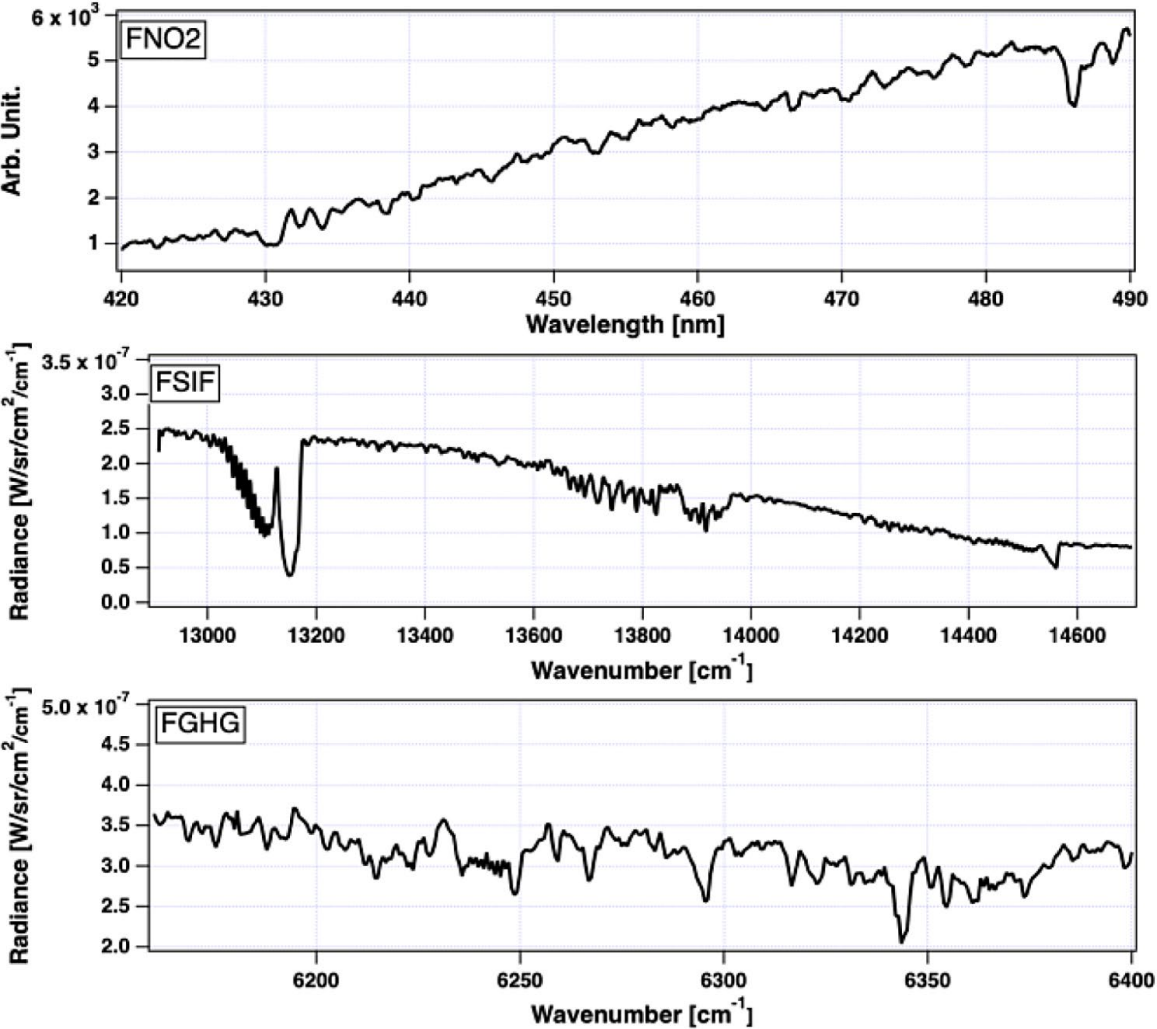
The collected spectra by FNO2 (top), FSIF (middle), and FGHG (bottom) during a ground function test period

Like passengers, these " passenger " instrument boxes are mounted on seats also with seatbelts. Thus, no modifications to the aircraft are needed. In theory, these instrument suites can take a cabin seat on either side of the aircraft; however, we must switch the setting depending on the side of choice and optimize the coordination. To avoid the missetting, the instrument suites are labeled as “-L” for left-side use and “-R” for right-side use. Figure 1 shows the instrument models for left-side seats. The typical spectra observed by the imaging suites, presented in Fig. 1, are also plotted in Fig. 2.

**Table 1:** A summary of instrument details

Instruments	FNO2	FSIF	FGHG
Spectral range	420–490 nm	670–780 nm	1560–1640 nm
Spectral bands	2048	2048	640
Spectral sampling interval	0.03 nm	0.05 nm	0.17 nm
Spectral resolution (FWHM)	0.8 nm	0.17 nm	0.3 nm
Spatial pixels	2048	2048	512
Bundled spatial pixels	53		
Field of view	31º		
Instantaneous field of view	0.58º		
Spatial sampling interval (along-track)	0.5 s		

The optical lens and optical fibers are made of quartz glass for wide-spectral coverage. Two packed instruments can be handily to mounted on a passenger economy (coach) class seat. The input optics, the passengers’ “eye”, combines a wide viewing lens and an inertial navigation unit, coupled with a Global Positioning System (GPS) signal receiver, to collect the solar radiation reflected by the Earth’s surface and identify the starting locations.

In addition, during taxing before and after the observation flight, a mechanical shutter shields the lens, and the dark spectra are acquired for reference. While the cabin pressure and temperature of passenger aircraft are well controlled, localized temperature gradients around the instrument suites should be avoided. Circulators are installed for each instrument suite. Both navigation and spectra data are simultaneously logged on a laptop computer through a USB-3.0 connection, and the collected data can be monitored in flight.

All the electronic power for the electronic devices, including camera image acquisition, is supplied by a mobile Li-ion battery (Fujikura BA-155, 155 Wh). The battery capacity needs to be less than 160 Wh to be on board an aircraft, according to a regulation by Japan’s Ministry of Land, Infrastructure, Transport and Tourism (MLIT). The power supply is installed in carry-on luggage-sized boxes, and this configuration is permissible on ANA flights. A single battery with the current observation configuration allows continuous NO_2_, SIF, and CO_2_ observation for at least three hours. For round-trip observation, these batteries can be switched with fully recharged packs at the airport, allowing an additional three hours of observations.

### The retrieval principles

To retrieving NO_2_, SIF, and CO_2_, the differential optical absorption spectroscopy (DOAS) technique [27], the iterative maximum a posteriori differential optical absorption spectroscopy (IMAP-DOAS) technique [28] and full physics retrieval technique [e.g. 29–37] are planned to apply, respectively.

The NO_2_ absorption lines allocated between 420 and 490 nm are applied DOAS spectral fit. To process the DOAS fit for NO_2_ retrieval from GOBLEU, the QDOAS software is optimized for GOBELU analysis [38]. The detail of NO_2_ retrieval setting and data analysis are described in results section.

Solar induced Chlorophyll Fluorescence (SIF) is emitted between 600 nm and 900 nm with the peak at 685 nm [39]. To observe the SIF with remote-sensing, the isolated Fraunhofer lines (absorption features in the solar atmosphere) are used to retrieve the fluorescence emission intensity from O2 A-band observed by GOSAT [40–44]. In addition, SIF is retrieved from GOME-2, TROPOMI [45–47] both O2 A-band and B-band. To cover the isolated Fraunhofer lines allocated both O2 A- and B-bands for SIF retrieval, our instrument cover between 670 and 780 nm with 0.05 nm spectral sampling interval. SIF emission intensity from the collected GOBLEU spectra is planned to retrieval by optimizing the iterative maximum a posteriori differential optical absorption spectroscopy (IMAP-DOAS) technique [28] both O2 A- and B- bands regions.

In the case of space-based CO_2_ observation, 1.6 μm (weak CO_2_ absorption spectra) and 2.0 μm (strong CO_2_ absorption spectra) of CO_2_ bands are used as CO_2_ retrieval channels among GOSAT [29–35], GOSAT-2 [36, 37], OCO-2 [48] and OCO-3 [48]. These retrievals are coupled with strong and week CO_2_ channels and O2A channels to detect the effective optical path length. The transmittance of cabin window only allows to transmit week CO_2_ spectra. The CO_2_ retrieval from GOBLEU flight will be performed with full physics algorithm, its constructed with the simultaneous spectral fit between the observed week CO_2_, O_2_ A-band spectra and theoretical radiative transfer calculation. Additionally, IMAP-DOAS for CO_2_ [49] is also considered to first processing of CO_2_ enhancement in the region of interest.

### Observation pattern

**Fig. 3:**
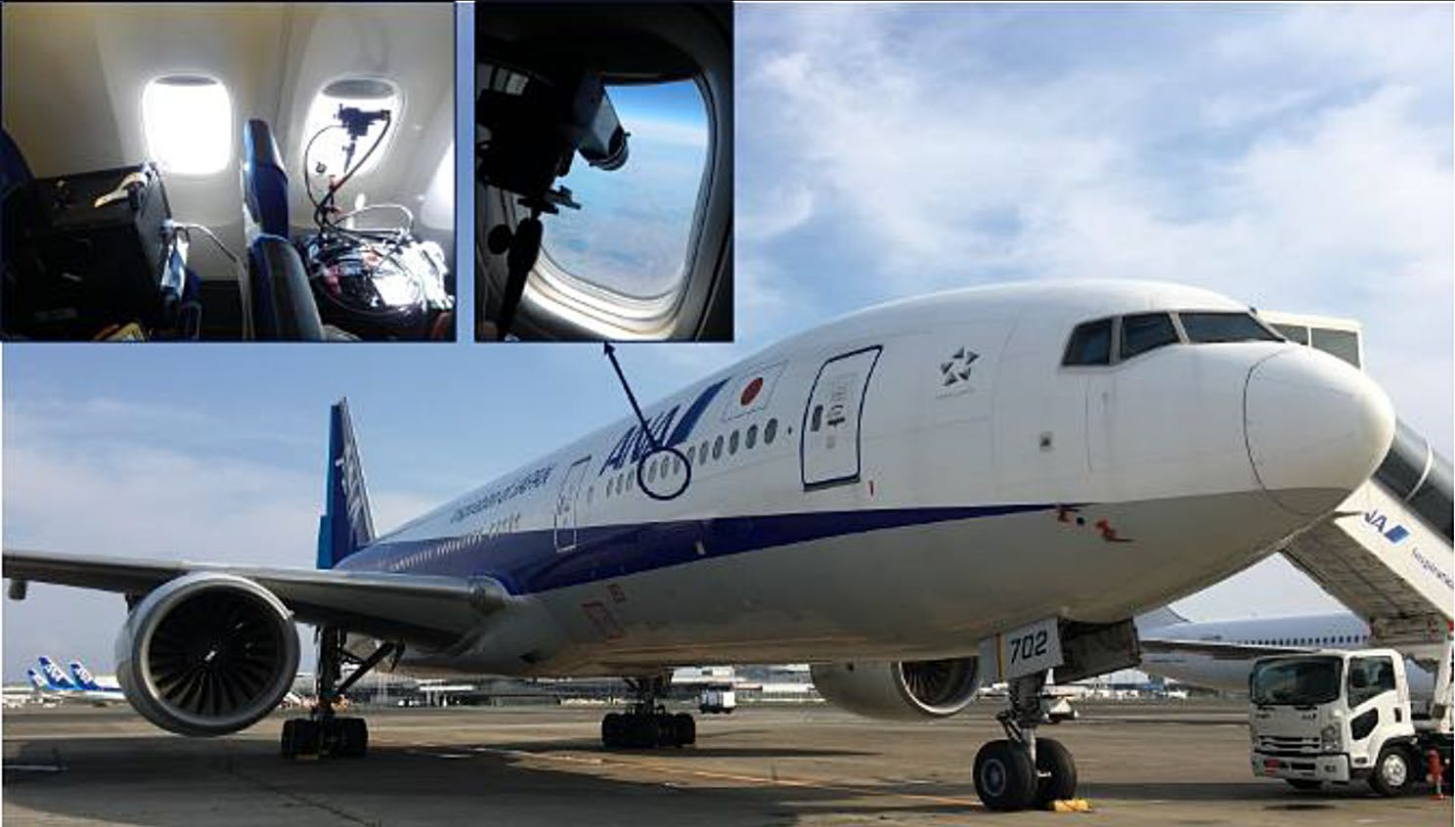
The GOBLEU instrument suite onboard the ANA aircraft. The instruments are seated on cabin seats (top left panel), targeting the input optics to slant-view thorough the cabin window (top right panel)

**Fig. 4:**
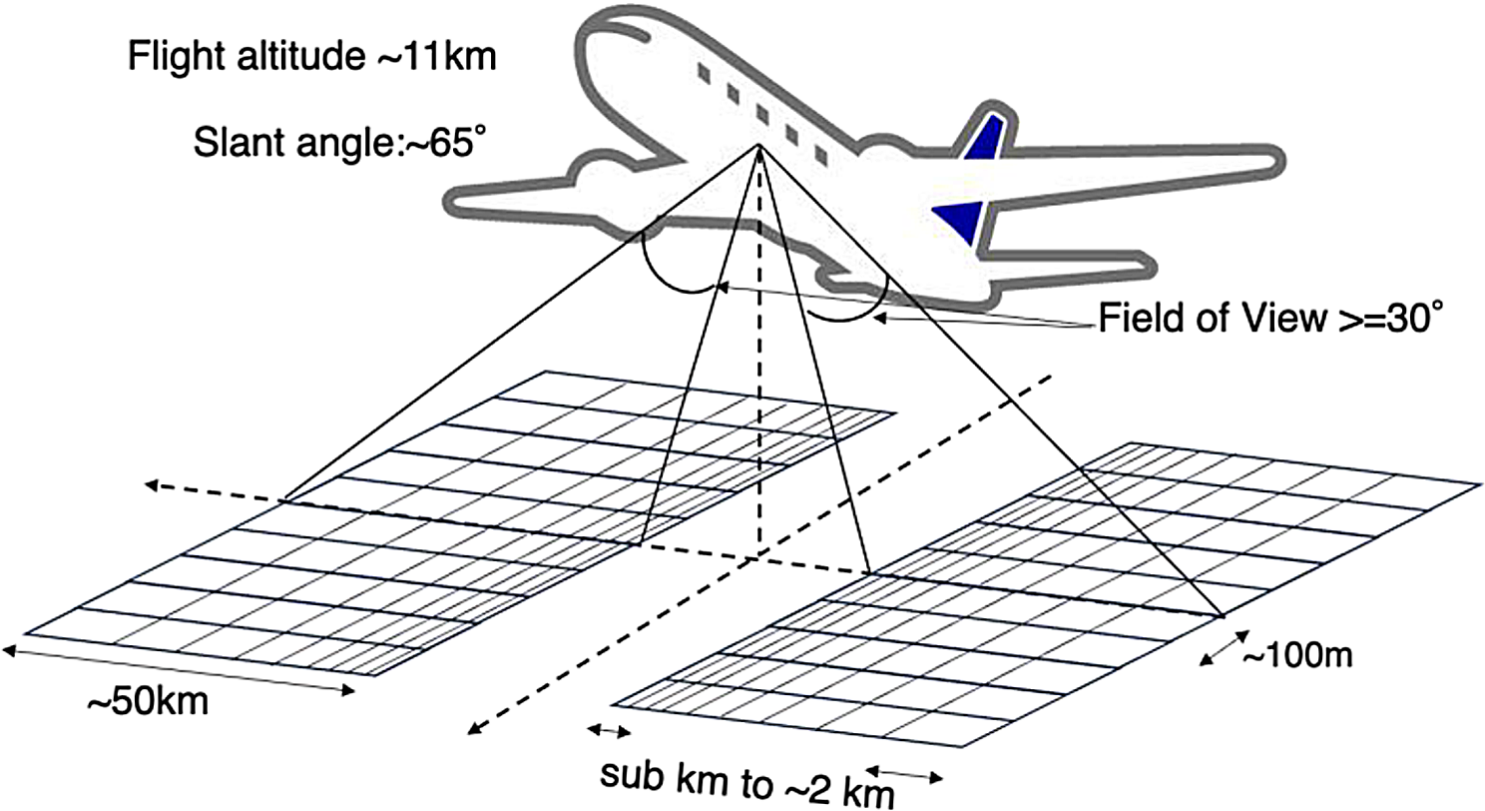
The GOBLEU observation configuration. “New passenger” (Monitoring instruments) onboard ANA passenger flights and slant-viewing to detect changes in NO_2_, SIF, and CO_2_ levels over the surface using cabin seats. A typical sampling distance in along-track is 100 m with 50 km across-track coverage. In the double-side case, the coverage is up to 100 km. The sampling size for across-track direction depends on the viewing angle from the aircraft, and it varies from sub-km to 2 km

The passenger’s eye views the slant nadir through the cabin window, where the observation viewing angle is managed around + 65 º from the nadir to avoid the vignetting of the solar light reflecting the Earth’s surface and stray light from the cabin window and its frame. The pictures of GOBLEU imaging spectrometer suites mounted on the seat of an ANA aircraft are presented in Fig. 3. This configuration can provide wide spatial coverage. From one side of the aircraft (as shown in Fig. 4), the spatial coverage is around 50 km with an 11 km flight altitude and + 65 º viewing angle. It directly depends on flight altitude and viewing angles. The spatial coverage can be easily doubled by expanding to two-side (right and left side of the aircraft) observation with additional carry-on equipment. The ground sampling distance (GSD) is 100 m (along-track) by sub km (across-track) assuming a typical altitude of 11 km a. g. l., ground speed of 200 m/s and integration time of 500 milli-seconds. This configuration will support an increase in monitoring frequency and spatial coverage. In the case of double-sides observation, the coverage for across-track is up to around 100 km with 100 m along-track spatial resolution, which is ten times higher than current satellites.

### Flight route plan

**Fig. 5:**
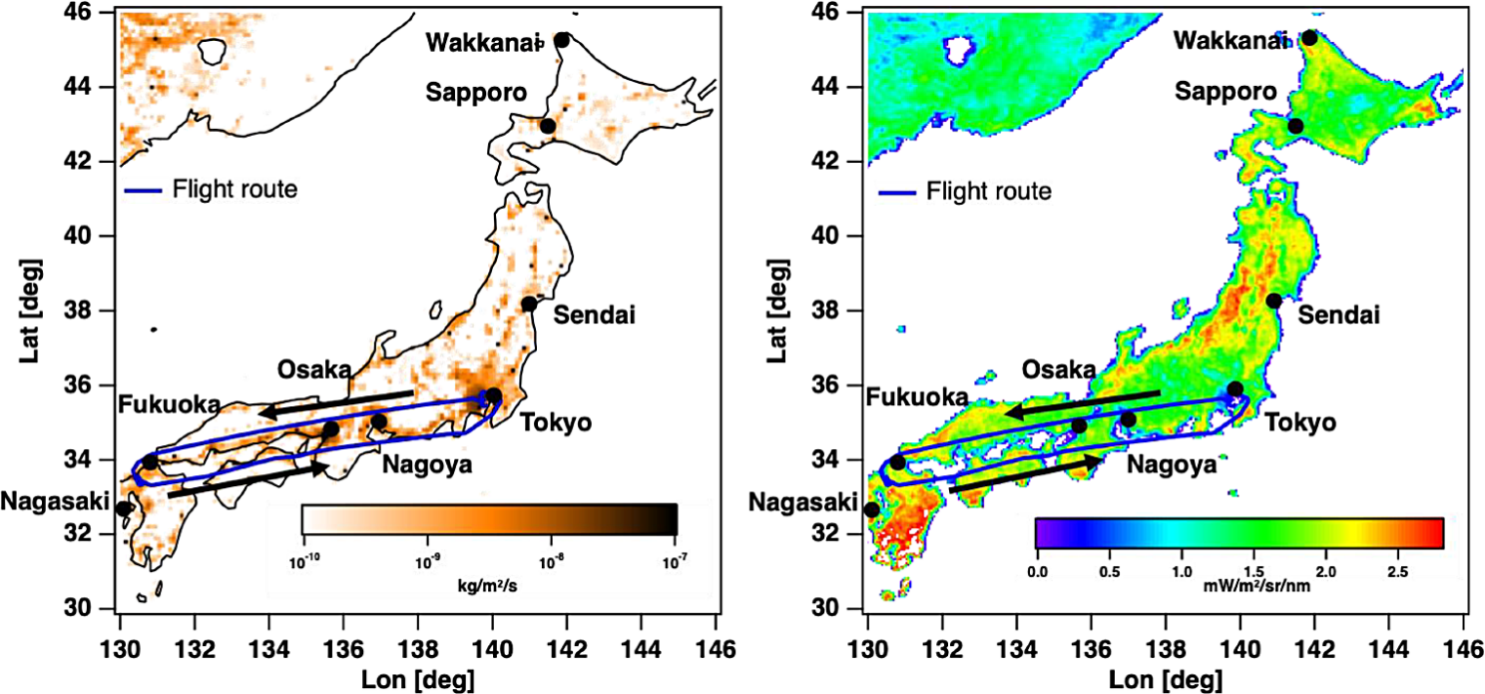
The observation flight route with an EDGAR CO_2_ emission map (left) and a TROPOMI SIF intensity map (right). Flight routes cover Tokyo, Osaka, Nagoya, Fukuoka, Sendai, and Sapporo in Japan and industrial area between Tokyo to Fukuoka

The ANA flight nationwide route network that connects Japan’s megacities should allow our observations to focus on collecting data over those intense local emission areas where current satellites have had a challenge to collect data. As described earlier, our operation can be extended to three hours without replacing a battery. We can select any direction from Tokyo Haneda Airport in this time frame. The longest leg is northward from Tokyo Haneda Airport to Hokkaido Wakkanai Airport, which takes three hours. Southward, a 2.5-hour flight from Tokyo Haneda Airport to Kyushu-Nagasaki Airport is allowed. These flights cover the major Japanese megacities and large industrial areas. The main observation route, from Tokyo Haneda Airport to Fukuoka Airport, is illustrated in Fig. 5 coupled with the Emissions Database for Global Atmospheric Research (EDGAR) CO_2_ [50–52] emission map (left) and the one-year integrated TROPOMI SIF intensity [53] (right). The left panel in Fig. 5 illustrates the Tokyo Haneda to Kyusyu Fukuoka flight route and CO_2_ emission strength based on EDGAR CO_2_. These routes cover Japanese megacities such as Tokyo (pop: 140 M), Osaka (pop: 88.2 M), Nagoya (pop: 22.9 M), Fukuoka (pop: 15.4 M), Sendai (pop: 10.8 M), and Sapporo (pop: 19.5 M). Also, the major industrial areas in Japan are often located in coastal areas, such as Tokyo Bay, Nagoya Bay, Osaka Bay, and Setouchi Bay. Regular passenger flights also cover these areas. Normally, it takes two hours from Tokyo Haneda Airport to Fukuoka Airport (the southern mega city in Japan). The two-hour flight allowed us to collect soundings every 0.5 s (around 0.8 million observations per side in two-hour flight; 53 across-track x 2 images/sec x 2 h) from 130°E to 140°E in longitude and 33.5°N to 36°N in latitude (about a 900 km travel distance).

## Results

### The first GOBLEU flight and data processing

On the 26th of October 2020, during the COVID-19 pandemic, the GOBLEU monitoring suite began its first trip from the Tokyo Haneda Airport to the Fukuoka Airport with an ANA regular flight (NH247). A summary of the first flight is shown in Table 2. This flight aimed to realize our observation concept; thus, only the NO_2_ module was activated. The main objective was to monitor NO_2_, CO_2_, and SIF via the cabin window using remote-sensing technology and operation on board a passenger aircraft. To focus on the proof of our observation concept (e.g., performed by one person for one instrument), the first observation test was performed using the NO_2_ instrument only with the minimum number of support staff. It also aimed to reduce the risk of Coronavirus infection during the COVID-19 pandemic.

**Fig. 6:**
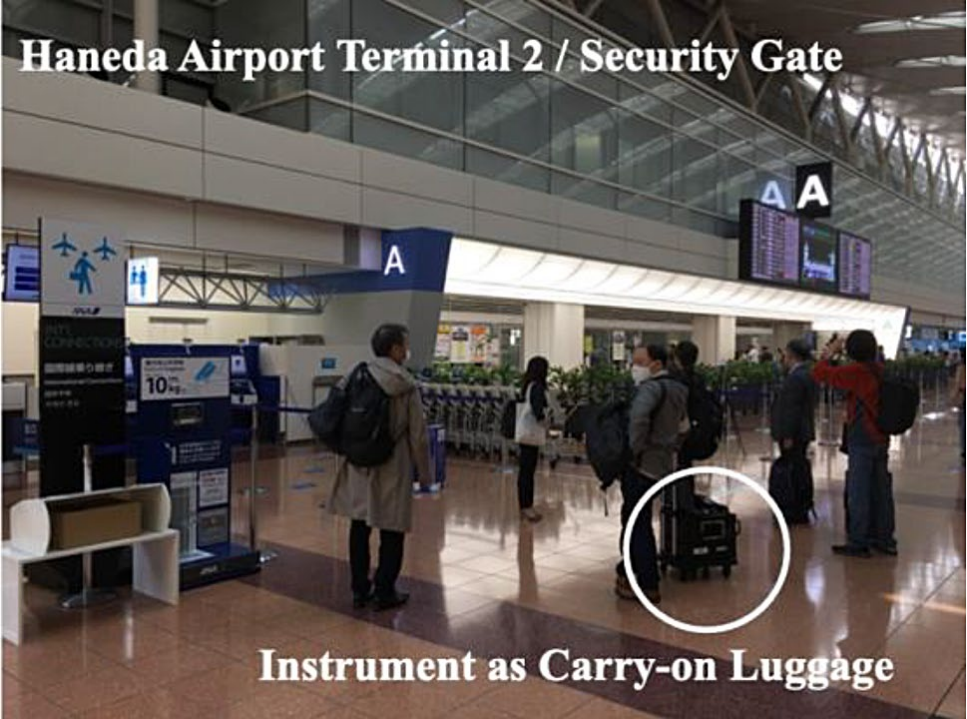
Carry-on instrument with operator. The picture was taken just before the security gate of Haneda Airport Terminal 2. The instrument is brought as carry-on luggage for the operator

The prototype instrument, with the same imaging spectrometer and input optics of the complete package shown in Fig. 1 but mounted on prototype packaging, was treated by an operator as carry-on luggage from the laboratory to Tokyo Haneda Airport (see Fig. 6). After passing through the security inspection including X-ray testing, the instrument was ready for the on-board commercial flight. The “passenger” instrument has been granted permission to be installed on the aircraft after conducting thorough radio interference checks and confirming with the airline that it will not cause any interference. With two pieces of carry-on luggage, any domestic observation flight from the Tokyo Haneda airport (central Japan) can be selected.

To secure the preparation time for the instrument on the cabin seat, we were preboarded ten minutes before regular boarding in the first trial. Then, the instrument was fixed with a seatbelt, as would be a typical passenger. As expected, the aircraft departed on time and took off smoothly. Once the captain turned the seatbelt sign off, we inspected the instrument and confirmed that there was no damage due to takeoff. During the flight, the operator constantly checked the computer display, which indicated two spectra in one second, real-time aircraft position and attitude, and file storage status. Also, the operator noted the weather conditions at several key locations, such as megacities and industrial areas.

**Table 2:** A summary of the first GOBLEU flight

Date	2020-10-26
Time	01:02–02:22 UTC and LT10:02–11:22
Location	From Tokyo Haneda Airport to Fukuoka Airport
Range	From 33.5ºN 139.8 ºW to 33.6ºN 130.4ºW
Aircraft (Aircraft Registration)	Boeing 767 − 300 (JA607A)
Flight Number	NH247
Observation Target	NO_2_
Solar zenith angle range	47.4º to 53.8º

The weather was fine for the first flight. The operator could observe the ground over Nagoya, Osaka, and the industrial area over Setouchi Bay. After 1.5 h from takeoff from the Tokyo Haneda airport, the aircraft landed at Fukuoka airport. Since the GOBLEU package was firmly mounted in the cabin seat, we did not observe any damage to the package during the landing. At Fukuoka Airport, the instrument suite was “relaxed” and unmounted and left the aircraft as the last passenger from the flight.

The collected spectra were analyzed by using the differential optical absorption spectroscopy (DOAS) technique to retrieve NO_2_ data. The DOAS spectral fit was done for the 430–460 nm, which is optimal for NO_2_ retrieval from GOBLEU using the QDOAS software.

In this spectral fit, the differential slant column densities (*dSCD*) are retrieved with a reference for DOAS analysis, which spectra acquired over the rural area (no emission sources). The retrieved slant column density of NO_2_ was converted to the vertical column density of NO_2_ to consider the airmass factor (*AMF*)

Based on the visual inspection of simultaneously acquired visible camera images by GoPro 8, the highly cloud contaminated areas and the optimal reference area (no emission sources area) are identified. NO_2_ absorption cross sections were subsequently fitted to the differential optical depth as well as O_2_-O_2_, H_2_O, O_3_, the pseudo ring spectrum interjacences. Spectrally slowly varying signatures were accounted for including a 3rd order polynomial in the fit. The spectral calibration is also conducted in QDOAS processing coupling with a high-resolution solar spectrum, implemented in QDOAS [54]. As for the instrumental line shape function, the error function, which is based on the convoluted box car and gauss function, is selected for this analysis.

The AMF is defined as the ratio between slant and vertical column of a trace gases. The AMF inferred from the radiative transfer model with geographic and atmospheric model parameters. In this study, the linearized discrete ordinate radiative transfer model: LIDORT [55] are employed with the climatological NO_2_ profile, which provided from chemical transportation model results of the Tropospheric Chemistry reanalysis version 2 (TCR-2) database [56], surface albedo was set to the monthly climatology data of Lambertian-equivalent reflectivity (LER) derived from TROPOMI [57]. For the aerosol, Aerosol optical thickness (AOT) at 500 nm is applied the constant value of 0.21 coupled with gauss distribution model at1.5 km peak height. For other parameters such as O_3_ vertical profile are used US standard atmosphere.

Through the optimal fitting by QDOAS, the NO_2_$\:{dSCD}_{obs}$, which is the difference between $\:{SCD}_{obs}$ from the observation spectra and $\:{SCD}_{ref}$ from the reference spectra, is derived and express the formulation in Eq. (1).

1 $$\:{dSCD}_{obs}={SCD}_{obs}-{SCD}_{ref}$$

To deriving the vertical column density ($\:VCD$) of NO_2_, the estimated $\:{dSCD}_{obs}$ are converted to $\:{VCD}_{obs}$ coupled with$\:AMF$ both observation ($\:{AMF}_{obs}$) and reference scene ($\:{AMF}_{ref}$), and the reference of vertical column density ($\:{VCD}_{ref}$) by applying the Eq. (2).

2 $$\:{VCD}_{obs}=\frac{{dSCD}_{obs}+{VCD}_{ref}\times\:{AMF}_{ref}}{{AMF}_{obs}}$$

The model parameters used $\:AMF$ in the calculation, such as surface albedo, vertical profiles of trace gases including NO_2_ and the aerosol scenario were kept constant throughout all observation points. These AMF calculations are conducted for each observation pixels both reference and target.

In this study, areas around 137.6$\:^\circ\:$ of longitude and 35.0$\:^\circ\:$ of latitude are selected as reference area and 1.35 × 10^15^ molec/cm^2^ is assumed as $\:{VCD}_{ref}$ reference concentration, which was determined using climatological NO_2_ dataset based on monthly averaged TROPOMI observation data [58]. The differential approach cancels out the stratospheric NO_2_ concentration to the signal, making the measurements only sensitive to tropospheric absorption, under the assumption that the stratospheric NO_2_ filed has a negligible spatial and temporal variability during the time between the aquation of the reference spectrum and the measurements.

The error in the retrieved NO_2_$\:{VCD}_{obs}$ originates from uncertainties in the calculated $\:{dSCD}_{obs}$, $\:{SCD}_{ref}$, and $\:{AMF}_{obs}$. One assumes that the contributing uncertainties are sufficiently uncorrelated as they arise from nearly independent steps. Base on Eq. (1), the error of the NO_2_$\:{VCD}_{obs}$ retrieval algorithm can be quantified based on the following error propagation method [59]:

3 $$\:{\sigma\:}_{{VCD}_{}}^{}=\sqrt{{\left(\frac{{\sigma\:}_{{dSCD}_{obs}}}{{AMF}_{obs}}\right)}^{2}+{\left(\frac{{\sigma\:}_{{dSCD}_{ref}}}{{AMF}_{ref}}\right)}^{2}+{\left(\frac{{SCD}_{obs}\times\:{\sigma\:}_{{AMF}_{obs}}}{{AMF}_{obs}^{2}}\right)}^{2}}$$

To estimate a typical $\:{\sigma\:}_{{VCD}_{}}^{}$, Eq. (3) is considered with our flight data. The error in the DOAS fit, $\:{\sigma\:}_{{dSCD}_{obs}}$, is a direct output of QDOAS for each fit. In parallel, $\:{AMF}_{obs}$ is calculated for each observation point coupled with modeled parameters. For the analysis areas including Nagoya and Osaka, the first term in the error estimation equation is calculated as 4.8 × 10^15^ molec/cm^2^. (only in blanket in the first term.)

The second error term originates from the estimation of the NO_2_ residual amount in the reference spectra ($\:{\sigma\:}_{{dSCD}_{ref}}$), which is determined as the standard deviation of reference observations fits. In this assumption, the second term is calculated as 5.9 × 10^15^ molec/cm^2^. The calculated third term ($\:{\sigma\:}_{{AMF}_{obs}}$) has to be estimated based on the variation of modeled parameters.

It is too important to take the sensitivity of various parameter to AMF into account; however, it is beyond the scope of this study. Thus, the reference values, which were reported by [59], are applied in this estimation. In the literature, the relative error of $\:{\sigma\:}_{{AMF}_{obs}}$ was based on” Heavily polluted” condition in [59]. In this case, $\:{\sigma\:}_{{AMF}_{obs}}$ is assumed to be 0.6. To apply this assumption, the third term in the bracket is 6.8 × 10^15^ molec/cm^2^. Finally, a typical $\:{\sigma\:}_{{VCD}_{}}^{}$ was estimated to be 1.0 × 10^16^ molec/cm^2^.

As shown in the following sections, we compared the retrieved NO_2_ results with other data, such as satellite NO_2_, a gridded emission inventory and ground-based in-situ observations.

### The observed NO_2_ at a glance

**Fig. 7:**
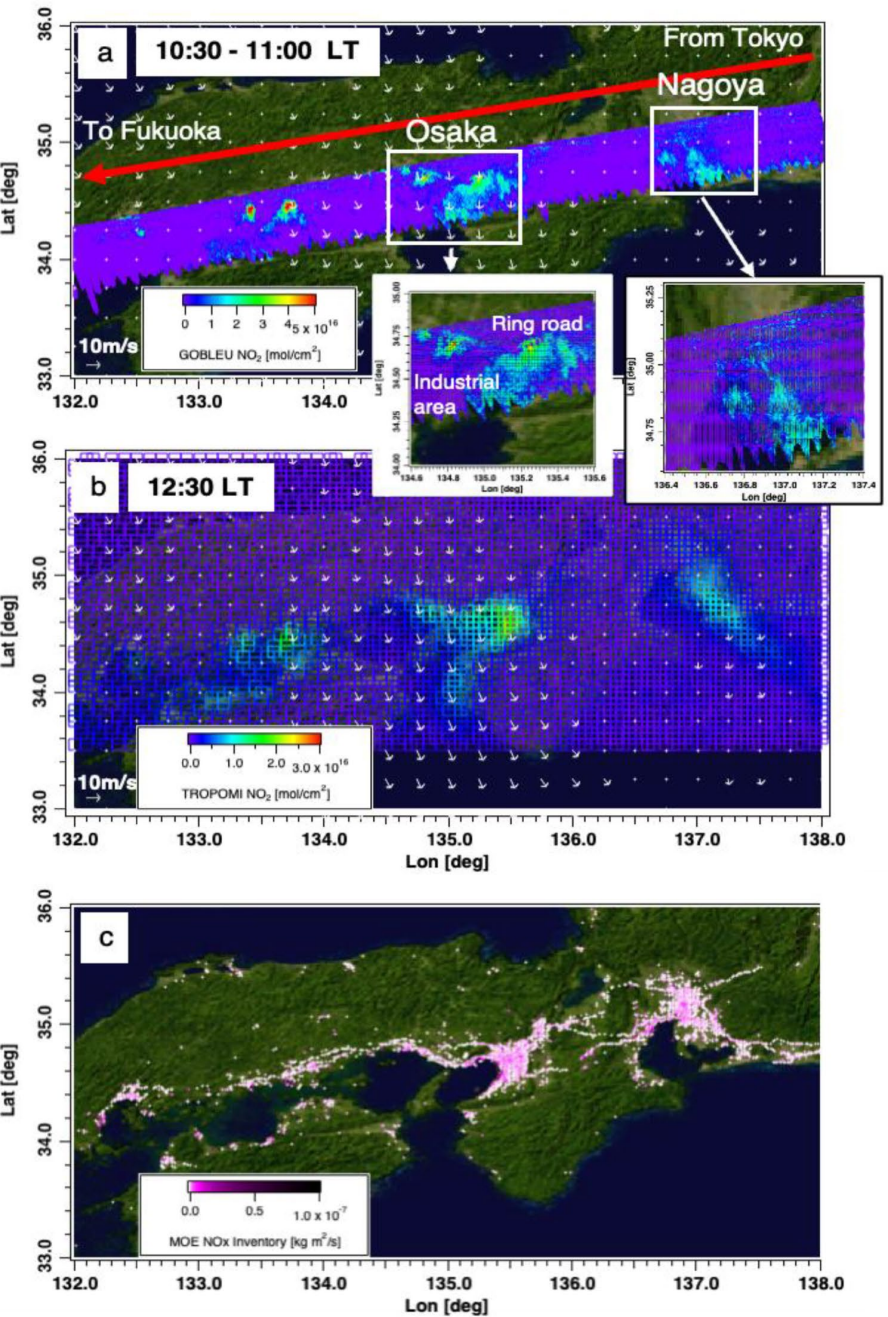
NO_2_ data collected during the first GOBLEU flight between Tokyo Haneda Airport and Fukuoka Airport (**a**) and from TROPOMI (**b**). The bottom panel (**c**) shows 1 km x 1 km total NOx emission values from the MOE inventory. The white arrows present the wind vector: GOBLEU for UT 10:00 and TROPOMI NO_2_ for UT 12:00, respectively

Figure 7 shows the collected first high-definition NO_2_ map (Fig. 7a) showed good spatial pattern correspondence between both NO_2_ data collected by the state-of-the-art satellite NO_2_ instrument TROPOspheric Monitoring Instrument (TROPOMI) [60, 61] (Fig. 7b) and a high-resolution (1 km) gridded NO_X_ total emission estimates (Fig. 7c) developed by the Ministry of the Environment (MOE) (hereafter, MOEJ) [62], notably at megacities, such as Nagoya (pop: 7.5 M) and Osaka (pop: 8.8 M). The observation duration for these areas is around 30 min for GOBLEU and less than 1 min for TROPOMI. The white arrows present the wind vector taken from the ERA5 hourly wind dataset (GOBLEU for UT 10:00 and TROPOMI NO_2_ for UT 12:00) [58, 63]. The map depicts the spatial pattern of NO_2_ over the two megacities. In addition, the emissions from industrial areas are also clearly captured. The general spatial pattern is consistent with what TROPOMI satellite NO_2_ observations captured approximately two hours after the GOBLEU flight.

**Fig. 8:**
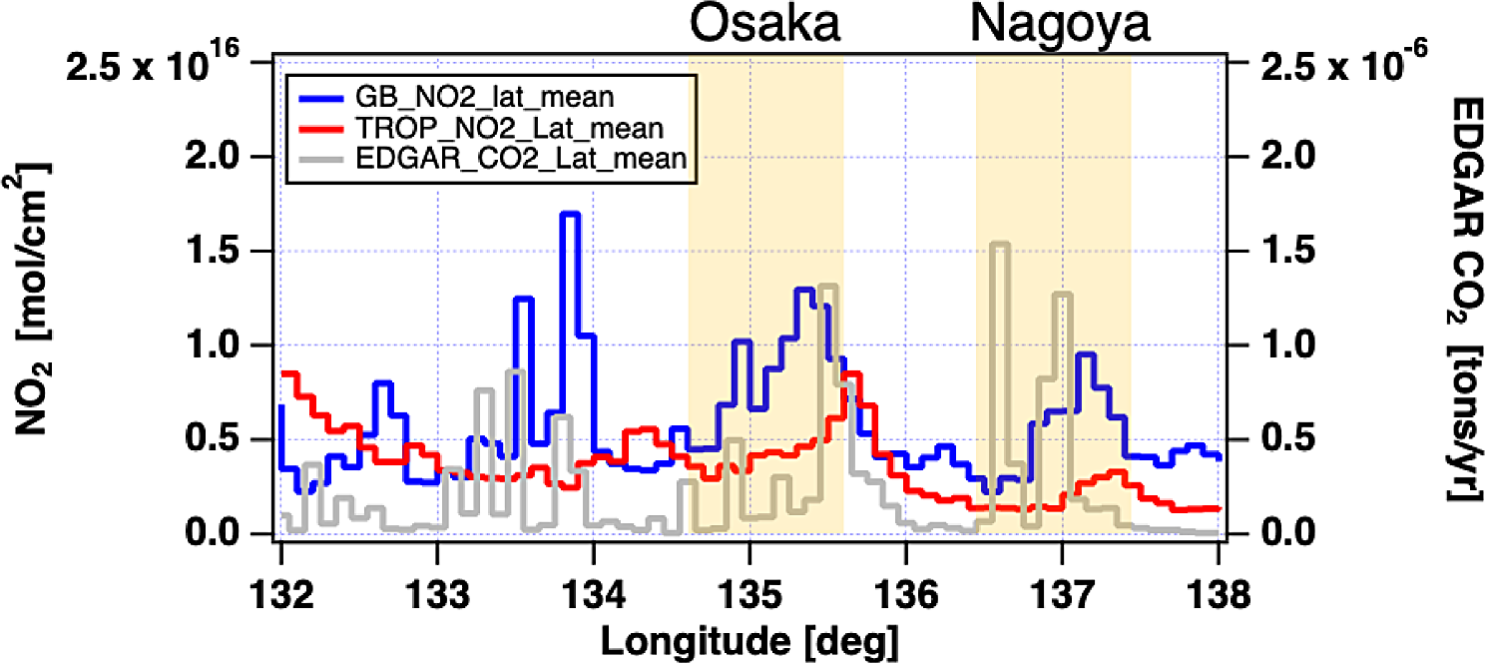
0.05º latitudinally averaged NO_2_ distribution of both GOBLEU (GB in blue) and TROPOMI (TROP in red) with EDGAR CO_2_ inventory (Gray). Yellow shading area indicates megacities’ longitudinal range. The Nagoya and the Osaka area are shaded

GOBLEU and TROPOMI NO_2_ spatial patterns are compared. Two NO_2_ datasets (34°-37°in latitude and 132°-138°in longitude) were binned at a 0.05° and averaged. The values were finally aggregated latitudinally. The GOBLEU NO_2_ map (aggregated mean in 0.05º longitudinal range, see Fig. 8) also showed good spatial correspondence with NO_2_ data collected by TROPOMI (aggregated mean in 0.05º longitude range). While the observations were taken two hours apart, GOBLEU and TROPOMI NO_2_ data shared major longitudinal spatial patterns peaking at megacity locations. It is important to note that GOBLEU data show more spatial features in the NO_2_ field that seem to be co-located with large CO_2_ sources of emission indicated by the EDGAR CO_2_ inventory [50–52]. EDGAR CO_2_ inventory data aggregated mean in the 0.1º longitudinal range is also plotted in Fig. 8.

### The NO_2_ spatial patterns over megacities

**Fig. 9:**
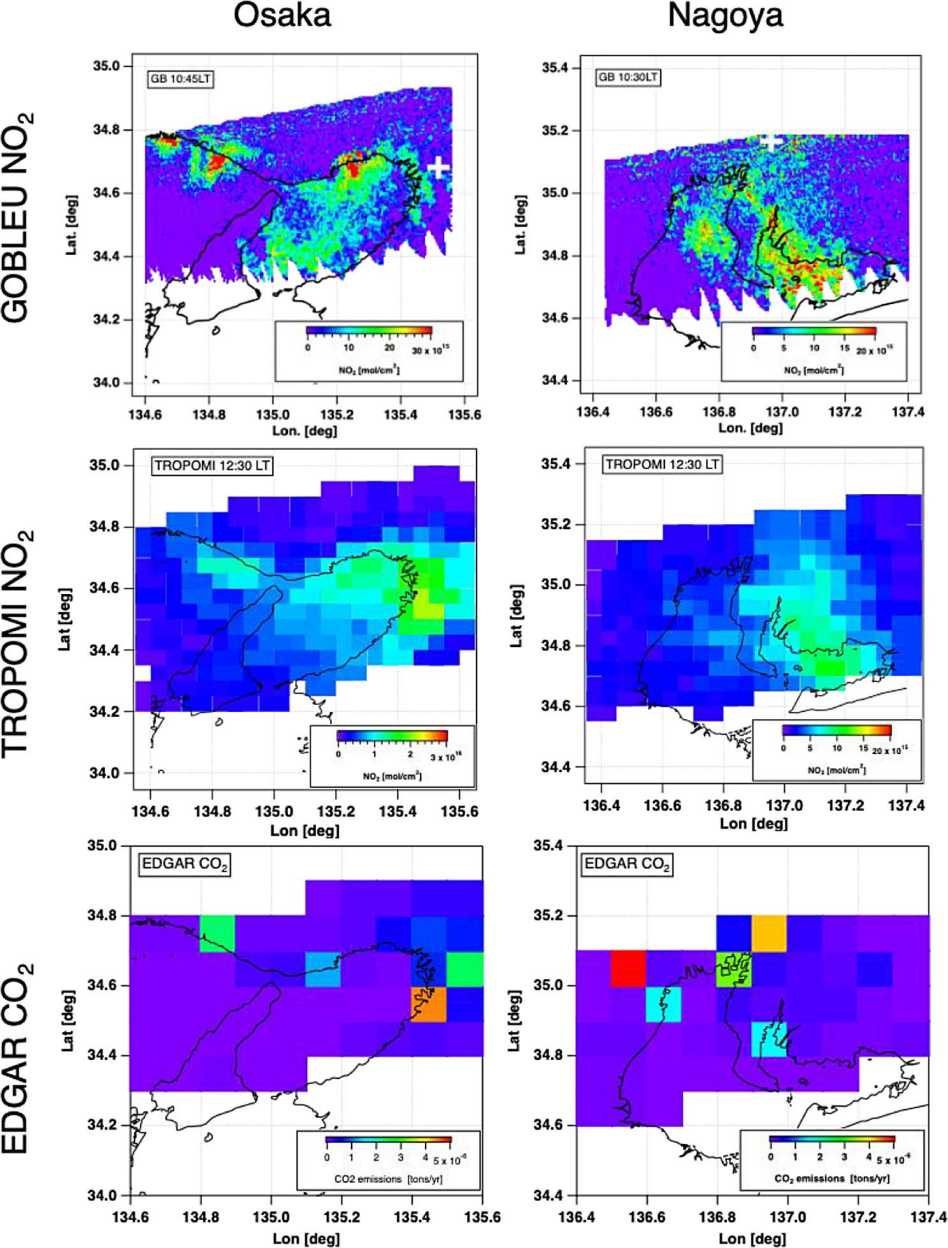
NO_2_ spatial patterns observed from GOBLEU (top) and TROPOMI (middle) during the first flight over Osaka (left) and Nagoya (right) during one flight. The boom row shows the spatial distributions of CO_2_ emissions from the EDGAR inventory. Note that the local times for GOBLEU and TROPOMI are not the same. While the two remotely sensed NO_2_ data share major spatial patterns, GOBLEU data captured fine-scale emission hot spots that might be diluted in TROPOMI data due to the spatial resolution

Looking closely at Nagoya and Osaka, the two maps showed different fine-scale spatial patterns. Figure 9 shows GOBLEU NO_2_ aggregated to 0.005º x 0.005º and TROPOMI NO_2_ aggregated in 0.05º x 0.05º with EDGAR CO_2_ aggregated in 0.1º x 0.1º. The GOBLEU NO_2_ map also depicts the point-wise intense NO_2_ concentration, which collocated with the power generation facilities. The differences are unsurprising because two observations were taken at different local times (10:30 for Nagoya and10:45 for Osaka). The observed NO_2_ concentration with different time presents the different concentration due to the diurnal cycles in emission and NOx-chemistry [64] as well as the wind magnitude/direction changes. However, they suggest the potential impact of observation time difference on the NO_2_’s performance as a marker for CO_2_ emissions and highlight the significant benefit of simultaneous CO_2_ and NO_2_ observations for accurately estimating CO_2_ emissions. Even a few hours’ difference in observation time (which we see in previous studies) could significantly impact our ability to estimate CO_2_ emissions using an NO_2_ marker. In addition, the high-resolution observation should help capture the activity level emissions signatures. GOBLEU provides 10 or 20 times finer spatial structures of NO_2_ concentrations than that of TROPOMI and the EDGAR inventory.

**Fig. 10:**
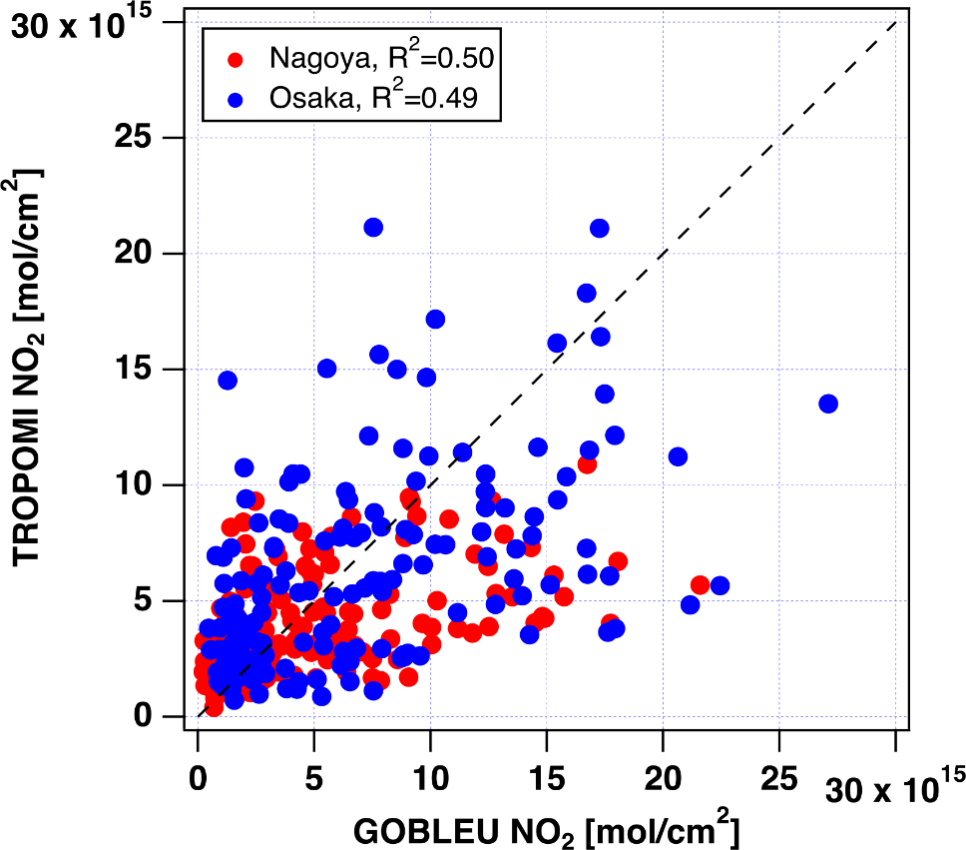
Comparison of GOBLEU NO_2_ and TROPOMI NO_2_ in Nagoya (red filled circle) and Osaka (blue filled circle). The dotted lines represent the 1:1 line. The range of variation between GOBLEU and TROPOMI is similar but GOBLEU NO_2_ present a higher concentration than that of TROPOMI. It seems that GOBLEU has highly sensitivity for local emission changes due to higher spatial resolution than that of TROPOMI

The spatial correspondence between GOBLEU and TROPOMI was further examined using the 0.05º aggregated data (Fig. 10). The difference could be attributable to the NOx-chemical process, wind speed and direction for NO_2_ transportation, and diurnal emission changes of NO_2_ due to the two-hour observation time difference, regardless of the potential contributing factors. The comparison shows that NO_2_ concentration derived from GOBELU is almost similar range with that of well validated TROPOMI NO_2_.

### Temporal correlation of NO_2_

**Fig. 11:**
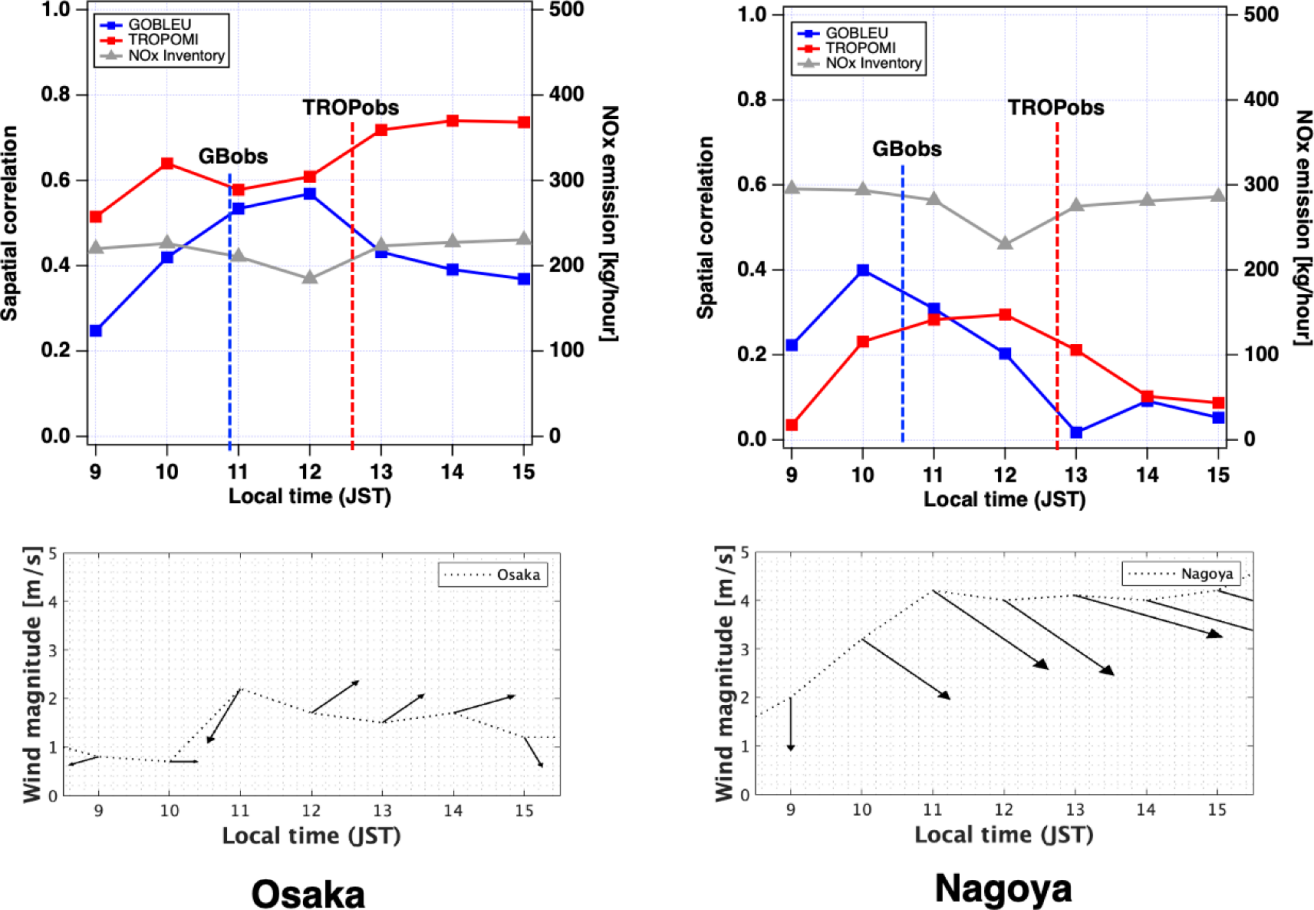
Correlation analysis using remotely sensed NO_2_ data from GOBLEU (GB in blue), TROPOMI (TROP in red), and hourly surface NO_2_ data. The spatial correlation of remotely sensed and surface NO_2_ was calculated over Osaka (top left) and Nagoya (top right) as a metric to characterize spatial patterns of two remotely sensed NO_2_ levels. Hourly changed wind magnitude and direction are plotted in bottom panels. The vertical dotted lines indicate the local observation times for GOBLEU (blue) and TROPOMI (red). The gray line shows hourly NOx emission estimates taken from a gridded inventory developed by MOEJ. The correlation tends to be higher near the local observation times for GOBLEU and TROPOMI; however, the two remotely sensed NO_2_ data collected roughly 1.5–2 h apart clearly showed a different correlation with the surface NO_2_ over time. This suggests the significance of collocated NO_2_ observations. The GOBLEU case showed much larger changes, which might be attributed to the higher spatial resolution of the data compared to TROPOMI

GOBLEU NO_2_ data’s performance as a CO_2_ emission marker was further examined by looking at spatial correlation with hourly NO_2_ data from ground-based monitoring sites (https://soramame.env.go.jp/station), acknowledging known challenges (see Fig. 11). Hourly data from 136 ground stations in Nagoya and 251 ground stations in Osaka are used for this analysis. These stations are designed to monitor major local emission sources within the cities, such as traffic and industries, mainly for air quality monitoring purposes. The correlation with these observational data should be able to evaluate the sensitivity of the remotely sensed data to local emissions.

As expected, we found that GOBLEU’s observations showed the best correlation at their local time (10:30 for Nagoya and 10:45 for Osaka). The level of the correlation went down significantly with time. Interestingly, while TROPOMI’s correlations with the ground-based data peaked at the observation time (12:30 JST) for Nagoya, the Osaka case showed two correlation peaks in the morning (10 a.m. local time) and afternoon (2 p.m. local time). While GOBLEU and TROPOMI share the basic remote-sensing principle, GOBLEU captures NO_2_ changes at the surface significantly better than TROPOMI. This is a caution for estimating emissions at a particular time of the day rather than obtaining an average emission over a certain period, using NO_2_ as an emission marker. This analysis only loosely examined the skill at representing spatial patterns of potential CO_2_ emissions. Accurately deriving emissions using these markers is yet another major challenge due to meteorology and chemistry.

## Discussions

The first proof of concept flight with a NO_2_ prototype instrument was successfully carried out. We proved and confirmed that our instrument collects science-quality useful spectra through the cabin window without modifying the aircraft. The airborne remote-sensing observation collects denser and finer spatial resolution GHG because the flight altitude of passenger aircraft (~ 11 km) is 1/60 of the current satellites (666 km) [3]. The follow-on instruments, a complete package of NO_2_, SIF, and CO_2_ modules, will be onboard both sides of the aircraft after the instrumental preparation and inspection. GOBLEU will simultaneously observe CO_2_ and SIF, in other words, the emission and removal makers. The SIF information as an indicator for an activity level of removals is informative to improve our knowledge of biospheric removals and provide this information.

We also plan to perform extensive evaluation and validation of the collected data using the upper-looking data collected by the Total Carbon Column Observing Network (TCCON) [65] and Collaborative Carbon Column Observing Network (COCCON) [66]. These observations are based on the sun-directed ground-based spectrometer. As for NO_2_, JAXA also prepared a ground-based instrument, PANDORA [67], for NO_2_ validation. In the future, we will coordinate the validation campaign for our datasets to set the ground-based instrument under the flight route.

**Fig. 12:**
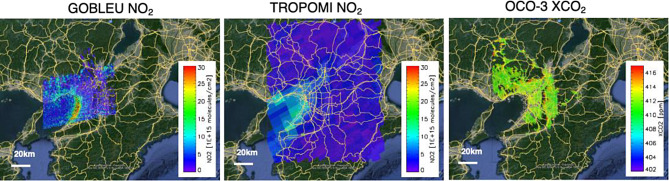
GOBLEU, TROPOMI, and OCO-3 images over Osaka. Emission proxy NO_2_ data over Osaka collected by GOBLEU NO_2_ (left, 13:15 LT; UTC + 9), TROPOMI NO_2_ (middle, 11:11 LT; UTC + 9), and OCO-3 CO_2_ (right, 11:05 LT; UTC + 9). These observations were conducted on the same day (Oct. 27, 2020.) but at different times. GOBLEU data depict the local pattern of NO_2_ concentration over Osaka. TROPOMI also suggests the high NO_2_ concentration area near Osaka Bay, but it is difficult to identify the individual sources by pixel. OCO-3 also indicated a high CO_2_ concentration area but did not correspond to the TROPOMI NO_2_ map. These results also encourage the simultaneous observation of CO_2_ and NO_2_, to understand the emission sources well

Our GOBLEU observation plans to prototype the synergistic use of CO_2_ and NO_2_ data and improve anthropogenic emission estimates. By using the correlation between CO_2_ concentration and SIF intensity, GOBLEU also aims to estimate the carbon removals in the surrounding megacity area by the terrestrial biosphere. By observing emissions and removals, GOBLEU aims to collect actionable data for monitoring emissions from dominant source sectors, such as local traffic and industries, within cities and the removal impacts by the terrestrial biosphere. By visualizing the emission strength and reduction impact by utilizing new emission reduction technologies, our mitigation effort will be clearly depicted on the map. As also described in the results section, GOBLEU data has fine spatial resolution and is more detailed than that of state-of-the-art spaceborne imaging spectrometer instruments such as TROPOMI, OCO-3, and new geostationary air quality instruments: Geostationary Environment Monitoring Spectrometer (GEMS) [68]. Then, GOBLEU data can potentially validate these spaceborne datasets with finer spatial resolution data. Figure 12 presents the spatial distribution comparison between GOBLEU, TROPOMI, and OCO-3. GOBLEU and TROPOMI data are aggregated in 0.005º x 0.005º, in 0.05º x 0.05º, respectively. OCO-3 has a 1.29 km x 2.25 km spatial resolution. Comparing these plots, GOBLEU data depict the local pattern of NO_2_ concentration.

While aircraft-based data are unique and valuable for GHG research [24], it is often challenging to increase data volume due to the cost. Our observations can be performed with two economy seats for instruments, the same as regular passengers. Taking a charter flight instead of a regular passenger flight increase the cost of one-flight up to several times. For example, two hours research flight could cost 2.4 K USD [https://www.jsforum.or.jp/other/zerog/]. Also, every day, passenger flights are scheduled to fly from one city to another, and seats can greatly increase the chance for observability. These situations are highly beneficial when applying for a regular passenger flight. The GOBLEU instrument has currently booked its seats on a regular flight, operated daily at noon (e.g., ten round-trip flights between Tokyo Haneda Airport and Fukuoka Airport from 9:00 JST to 17:00 JST). If we select multiple flights in a day, GOBLEU could be a “frequent flyer” to identify the diurnal variations. As described in the previous section, all the security and safety inspection including X-ray test, radio interference checks and confirming with the airline that it will not cause any interference are required and confirmed for GOBLEU. However, operator training will be required to install each module safely onboard. After optimizing the FGHG, FNO2 and FSIF instrumental package, regular observations once per month were started in the spring of 2023. During the COVID-19 pandemic, the seats for instruments were easily bookable. However, after the pandemic, the regular passenger returns on their flight, and the instruments must book months in advance. In this operation chain, selecting a flight with fine weather is difficult. To overcome this, frequent flights with instruments are desirable with simple operation. Also, there are regular flights between Tokyo Haneda Airport and Fukuoka Airport less than every hour, from early morning to late night every day. In addition, GOBLEU should have a chance to elucidate diurnal variations and weekly dependent emission patterns with high-frequency observations.

As indicated in Fig. 5, ANA has a nationwide flight route network that connects key capital and major airports in Japan, such as Tokyo Haneda Airport and Hokkaido Wakkanai Airport, Tokyo Haneda Airport, and Kyushu Nagasaki Airport, which are potential destinations for future GOBLEU flights. These routes cover the high-intensity SIF, which indicates the production level (carbon removal) of the terrestrial biosphere area, such as the near-Sendai forestry area (see Fig. 5). The intensity of SIF indicates the strength of emission remover.

The area under the flight route Tokyo Haneda Airport to Fukuoka Airport includes approximately 30% of Japan’s total CO_2_ emissions and about 59% of Japan’s Gross National Product (GDP) [69]. However, it is important to note that our airborne remote-sensing approach also shares the challenge due to cloud contamination. In the satellite observation case, around 80% of the data is cloud-contaminated despite the small foot-print size [70]. The data yield is expected to be impacted significantly in the summertime. In addition to anthropogenic CO_2_ emissions, monitoring of natural CO_2_ emissions and removals is a key to Japan’s GHG management [71]. 67% of Japan’s national land area is cover the forest [72].

## Conclusion

Under the GOBLEU project, JAXA has developed a remote-sensing technique that can be operated on commercial passenger aircraft, by JAXA and ANAHD. The GOBLEU monitoring instrument suite is designed to collect CO_2_, NO_2_, and SIF data. The first trial flight with the NO_2_ instrument was carried out and collected NO_2_ data over major populated and industrialized areas. The NO_2_ data showed timely snapshots of NO_2_ spatial distributions, suggesting the utility of NO_2_ data as a proxy for anthropogenic CO_2_ emissions that should enhance emission estimations and attribution ability. GOBLEU instrument can be operated in almost any passenger aircraft without any modifications. While based on the same space-based observation technique as the current satellites, our airborne observation can collect denser and higher spatial resolution GHG data more frequently over key emission areas, such as Japanese megacities.

GOBLEU is a new challenge for GHG remote-sensing observation on a new platform and has the potential to open up a new field for passenger aircraft use. Nominally, passenger aircraft only carried passengers. To contribute to climate action by air company, the ANA Group is promoting ESG management that considers the Environment, Society, and Governance, aiming to realize a sustainable society and enhance corporate value. Under the slogan of “ANA Future Promise”, the ANA group started actions to reduce the carbon emissions from aviation, actively adopting Sustainable Aviation Fuel (SAF) for aviation as well as a recycle-based society. In addition to these activities, ANAHD is scoping extended aviation use for climate mitigation activities such as climate monitoring tools.

GOBLEU aims to monitor the climate mitigation effort over Japan’s intensive industrialized areas and contribute to the second GST scheduled in 2028. GOBLEU expects to provide timely GHG information by promptly collecting high-resolution GHG data and emission and removal estimates with greater information granularity. The high-spatial-resolution data should provide GHG information to stakeholders at different subnational levels (e.g., states/prefectures, cities, private sectors, and citizens) toward carbon neutrality under the Paris Climate Agreement. We also expect to expand the observation coverage overseas with domestic and international partnerships through enhancing internal cooperation.

## Data Availability

All the data used in this manuscript are available from the following sources. EDGAR data: https://edgar.jrc.ec.europa.eu. OCO-3 data: https://www.earthdata.nasa.gov. The PM2.5 inventory data developed by MOE: https://www.env.go.jp/air/osen/pm/info.html. Soramame data: https://soramame.env.go.jp/download. TROPOMI NO_2_ data: https://scihub.copernicus.eu. TROPOMI SIF data: https://climatesciences.jpl.nasa.gov/sif/. GOBLEU data supporting this study are available upon reasonable request. Please contact suto.hiroshi@jaxa.jp. Snapshot images of GOBLEU data are available from https://www.eorc.jaxa.jp/GOSAT/ANAexp/index_e.html.
